# Evaluation of the nutritional value of fermented pangasius fish waste and its potential as a poultry feed supplement

**DOI:** 10.14202/vetworld.2025.355-366

**Published:** 2025-02-17

**Authors:** Abun Abun, Denny Rusmana, Kiki Haetami, Tuti Widjastuti

**Affiliations:** 1 Department of Animal Nutrition and Feed Technology, Padjadjaran University, Sumedang-West Java, Indonesia; 2 Department of Fisheries, Padjadjaran University, Sumedang-West Java, Indonesia; 3 Department of Animal Production, Padjadjaran University, Sumedang, West Java, Indonesia

**Keywords:** amino acids, bioconversion, enzyme activity, fatty acids, fermented fish waste, poultry feed supplement

## Abstract

**Background and Aim:**

The increasing global demand for sustainable and nutrient-dense poultry feed necessitates innovative approaches to utilize byproducts such as pangasius fish waste. This study explores the potential of bioconverted fermented pangasius fish waste (FPW) produced through microbial fermentation as a poultry feed supplement.

**Materials and Methods:**

The study was conducted in two stages. In the first stage, bioconversion of pangasius fish waste utilized a microbial consortium (PaRmYl: *Pseudomonas aeruginosa*, *Rhizopus microsporus*, and *Yarrowia lipolytica*) at varying inoculum doses (5%, 10%, and 20%) and fermentation durations (2, 4, and 8 days). Nutritional content, enzyme activity, and antioxidant properties were analyzed. The second stage involved biological testing on 90 broiler chickens (randomized into three treatment groups with 30 replications each) to assess digestibility and nitrogen retention of FPW-based feed.

**Results:**

Fermentation with a 10% inoculum dose over 4 days yielded the optimal nutritional composition, with crude protein content increasing to 37.27%, enhanced amino acid (EAA/NEAA ratio: 0.88), and fatty acid profiles (notably ω-3 and ω-6). Protease and lipase activity peaked at 1.49 U/mL and 1.21 U/mL, respectively, with antioxidant activity showing an IC50 value of 39.84 ppm. Biological tests demonstrated significantly higher dry matter digestibility (75.53%) and nitrogen retention (75.53%) in broilers fed FPW compared to non-fermented feed.

**Conclusion:**

FPW, produced through microbial bioconversion, offers a sustainable and cost-effective poultry feed supplement, enhancing digestibility and nutrient retention while addressing environmental concerns related to fish processing waste.

## INTRODUCTION

In ecotrophy and nutrition studies, white meat includes chicken and fish but excludes mammalian meat. The increasing demand for white meat underscores the need for sustainable and nutrient-dense feed alternatives. Therefore, it is crucial to increase fish and poultry production. Therefore, there is an urgent need for animal feeds that contain quality protein and lipids, one of which comes from fishery waste. Fishmeal has recently been questioned due to its low protein content, the impact of overfishing, and competition for use. On the other hand, with the increase in fish consumption, the frozen food sector, and the export of fish filets, it is conceivable that fish waste materials will become alternative feed materials. The potential demand for white pangasius meat, one of which comes from the *Pangasius* sp. (*djambal*) Bleeker species, is considerable and continues to grow [1]. Pangasius filets contain 45% meat, 24%–27% head residue, 12% skeleton, 3% skin, 4% cuts, and 12% intestines of total body weight [2]. Fillet processing produces a lot of waste from filet pieces such as skin, head, innards, bones, and fins, which typically vary between 20% and 60% from fresh fish, adding to the accumulation of waste worldwide [3]. Solid waste from fillet processing pieces contains high protein and fat. If not managed effectively, it will cause pollution due to protein decomposition or autolysis processes, and non-nitrogen compounds produce damaged protein fragments such as H_2_S, ammonia, indole, skatole, and others [4, 5]. Chemically treated fish silage waste, which is reported as an alternative solution [6], has environmental consequences such as 30% chemical emission waste, and according to Ghaly *et al*. [7], the nutrient content of chemical fish silage liquid products is generally lower than that of the original materials. Several studies have been conducted on the processing of fish silage from waste filets and market residues, including the chemical breakdown of fish proteins in an acidic solution into smaller units or processing by freeze-drying with the addition of NaOH to remove fat [7]. The primary purpose of making fish silage is to preserve it because animal waste materials have protein and fat contents that are susceptible to degradation and oxidation. As a result, it is crucial to investigate effective bioconversion techniques of microorganisms into fermented pangasius fish waste (FPW) to provide the necessary nutrients for animal protein feeds, mainly poultry feed. Existing fish silage solutions have limitations in terms of protein preservation and nutrient retention. This study investigates a microbial consortium to address these challenges.

Microbial fermentation is a natural and economical way to increase the value of feed ingredients by modifying their physical, chemical, and functional properties. Microorganisms are essential in the manufacture of healthier and safer fermented fish products because of their different metabolic features. Bioconversion through microbial fermentation provides biofuels, foods, and medicines [8, 9] and feed [10, 11]. The microorganisms in question include those added as starter agents and those found during fermentation [12]. Microbial enzymes and internal protease substrates have catalytic properties that are sensitive to dosage and fermentation time and are more stable at acidic pH [13]. *Pseudomonas aeruginosa* is a potential probiotic candidate for protection against *Staphylococcus aureus*, with enzymes produced on acidic media can accelerate its activity [14]. Fermentation of *P. aeruginosa* with 15% molasses starter for 15 days at room temperature (25°C) increased the crude protein (CP) content of fish waste from 58.17% to 64.15%. It slightly increases the indispensable amino acids Ca and P, but the dry matter (DM) decreases [15]. Process technology is required to produce amino and fatty acids that can be used as feed supplements. The biological fermentation process efficiently increases nutrient content while reducing anti-nutrient elements from various by-products and industrial waste. Fermentation products (FPW) mainly contain functional ingredients such as live probiotic bacteria and microbial metabolites. Alternative protein sources from fermentation also give rise to microbial proteins that synthesize high-value bioproducts through biowaste recycling as part of an innovative circular economy. From previous research, bioconversion by microorganisms is preferred because it is relatively inexpensive and involves simple artificial technology [15, 16]. A variety of probiotic microbes have been reported to be able to synthesize amino acids and fatty acids through proteolytic fermentation and lipolysis, including the species *P. aeruginosa, Rhizopus microsporus*, and *Yarrowia lipolytica* (PaRmYl), which are autochthonous bacteria in meat and fish [17]. Fermented products with probiotics are more advantageous with multistrain [18]. *R. microsporus* is a very potent proteolytic activity bacterium and can produce extracellular peptidase enzymes in a variety of fermentations [19]. *R. microsporus* is a safe microorganism, in addition, volatile acids and lactate R. microsporus increase protein breakdown activity and are more effective than *L. plantarum* 8014 and *B. subtilis* [10, 17]. Regarding the use of *Y. lipolytica*, it has a flavor-forming agent and can be a starter that utilizes a variety of ingredients [20]. Products with bioconversion contain functional ingredients such as live probiotic microbes and microbial metabolites. Alternative protein sources from fermentation also cause microbial proteins that synthesize high-value bioproducts through biowaste recycling as part of an innovative circular economy [21]. Probiotics for fermentation are more advantageous when used in a consortium with multiple strains; the addition of yeast *Y. lipolytica* will be more effective for its use in fermentation [22] The consortium can enhance the production of metabolites during fermentation due to the capabilities of all three types of probiotic microbes. Peptide production media can have a positive impact on bacterial growth and can form peptides through an enzymatic process [10, 23]. Fermentation produces value-added feed products such as proteins, oils, amino acids, minerals, enzymes, and peptides [24]. It is necessary to consider the factors that affect protein hydrolysis, namely, the dose, temperature, time, and presence of additives.

Improving feed quality using FPW bioconversion can increase poultry productivity. Good quality poultry feed contains nutrients that are suitable for the needs of the animal’s growth stage while also considering the balance of protein and metabolic energy of the feed. Balanced nutritional components in feed can promote optimal growth. In addition, including phytobiotics in the diet improves the balance of nutrients, which accelerates metabolic rate. Functional feed produced by a consortium of *P. aeruginosa, R. microsporus*, and *Y. lipolytic* seeks to increase the growth and production of eggs in poultry. Protein components, especially amino acids, glycine, and the mineral Fe, are expressed as hemoglobin constituents [25]. Oxygen and the number of erythrocytes in the blood both affect hemoglobin levels. When oxygen levels in the blood decrease, the body is stimulated to produce more erythrocytes and hemoglobin. Stressful conditions can disrupt several physiological effects, including blood profile and pH in the small intestine, affecting the digestibility of nutrients in the digestive organs during metabolism. FPW by the consortium of *P. aeruginosa, R. microsporus*, and *Y. lipolytic* can improve poultry digestibility and overcome the effects of stress in chickens. Based on the above statement, this study aimed to analyze the chemical composition, amino acid content, fatty acid profile, enzyme activity, and antioxidant activity of FPW produced by the consortium of *P. aeruginosa, R. microsporus*, and *Y. lipolytic*. Furthermore, the best products are scientifically evaluated using broiler chicken test animals to determine their potential as a poultry feed supplement.

## MATERIALS AND METHODS

### Ethical approval

The study was conducted on pangasius fish waste so, ethical approval was not necessary.

### Study period and location

The experiment was conducted from June to November 2023 at the Poultry, Non-Ruminant, and Animal Feed Industry Nutrition Laboratory, Faculty of Animal Husbandry, Padjadjaran University.

### Research stages

The research was divided into two parts; the first was to evaluate FPW bioconversion products based on nutrient content, enzyme activity, and antioxidant levels. The selected FPW products were then subjected to chemical testing followed by biological testing, which included digestibility and nitrogen retention (NR) in broiler chickens.

### The fermentation process and chemical tests

#### Materials

Research tools included meat knives, blenders, steamers, thermometers, autoclaves, laminar airflows, incubators, needle loops, Petri dishes, test tubes, micropipettes, glass slides, hot plate centrifuges, cuvettes, 50 mL volumetric flasks, ovens, 0.2 m GHP/RC syringe filters, high-performance liquid chromatography (HPLC), and 20 mL headspace bottles.

The material used was FPW, which consists of a mixture of fish heads, fins, innards, and bones. Microbes were inoculated with MRS (Mann-Rogosa-Sharpe), nutrient broth, trypticase soy agar (TSA), nutrient agar (NA), CaCO3, skim milk, hydrogen peroxide solution, oxidase strip, 2% casein solution, 8-pH phosphate buffer solution, 7-pH phosphate buffer, standard protease enzyme, standard lipase enzyme, aquadest, and alcohol (Merck KgaA, Jerman). *P. aeruginosa, R. microsporus, Y. lipolytica* (American Type Culture Collection, USA), and physiological NaCl 0.85%. The CP content was determined by the Kjeldahl micro method [26], using HCL 0.02 N, K_2_SO_4_, HgO, H_2_SO_4_, NaOH, Na_2_S_2_O_3_, and H_3_BO_3_. Fatty acid analysis was performed using E-penwork tubes, microliter micropipettes, and gas chromatography-mass spectroscopy. The amino acid analysis of fermented products was carried out by laboratory technicians of PT. Saraswanti Indo Genetech, Bogor-Indonesia (2023).

The antioxidant test was performed by preparing a 100 ppm 2,2-diphenyl-1-picrylhydrazyl (DPPH) solution by dissolving 5 mg of DPPH crystals in 50 mL of PA ethanol solution and storing it in a tightly closed container protected from sunlight. The sample was weighed as much as 10 mg and dissolved in 100 mL of PA ethanol, then diluted to several concentrations of 10, 20, 30, 40, and 50 ppm. A total of 10 mg of Vitamin C 1000 was weighed and then diluted with 100 mL of PA ethanol. The test solution was then placed into a test tube as much as 3 mL and added with 1 mL of DPPH, followed by incubation at a temperature of 27°C. During the reduction process by antioxidants, the radical DPPH solution changes from purple to pale yellow. This decrease in absorbance was measured using an ultraviolet spectrophotometer (Spectroquant Prove 600) at a wavelength of 515 nm (Merck KgaA, Jerman, Indonesia).

### Fermentation test stage

The manufacture of the inoculum was based on the results of previous studies [10, 27]*. P. aeruginosa*: Prepared in 125 mL Erlenmeyer flask containing 50 mL of sterile broth solution (adjusted to pH 7 using 1N HCl), and added pure and mineral culture solutions (yeast extract 0.5% (w/v), 0.5% (w/v) NH_4_NO_3_, 0.05% (w/v) KCl, 0.05% (w/v) MgSO_4_, 0.01% (w/v) FeSO_4_, and 0.001% (w/v) SePO_4_ then incubated (2 days at 45°C). *R. microsporus*: Prepared in broth solution medium, such as *P. aeruginosa* culture, then incubated for 3 days at 45°C. *Y. lipolytica* starter inoculum with broth solution incubated for 3 days at 30°C and prepared a mineral solution consisting of yeast extract 0.5% (w/v), 0.5% (w/v) NH_4_NO_3_, 0.05% (w/v) KCl, 0.05% (w/v) MgSO_4_, 0.01% (w/v) FeSO_4_, 0.001% (w/v) SePO_4_, and 0.5% (w/v) CaCO_3_.

### Bioconversion of FPW

Preliminary experiments were conducted to establish the dose and time of bioconversion based on the growth curve of PaRmYl microbes in catfish waste media. The results of the preliminary experiment indicated a minimum dose of 5%, an optimal dose of 10%, and a maximum dose of 20%. The bioconversion time should be a minimum of 2 days, an optimal of 4 days, and a maximum of 8 days.

The primary inoculum was diluted using 10-1 mL aquadest to obtain doses in percent volume per weight (PV/W), i.e., 5% (v/w), 10% (v/w), and 20% (v/w), which consisted of a mixture of three types of microbial inoculums. A meat grinding machine model precision 22S, 1.25 PK (Pendulum Kinematic) (Phywe, Jerman), cylindrical with a diameter of 30 mm and a length of 30 mm, was used to grind FPW. The grinding of each experimental unit was weighed, placed in an airtight 3-L jar, and code-numbered based on the treatment. Bioconversion of FPW by adding a secondary inoculum solution using a consortium of three PaRmYl microbes (*P. aeruginosa, R. microsporus*, and *Y. clipolytica*) according to treatment at room temperature (35°C–40°C).

### Research Design

Experiments were conducted to determine the effective dosage and fermentation time for producing the best FPW essential nutrients (CP content, crude fat, crude fiber, Ca, and P, as well as amino acid and essential fatty acid content). Then, enzyme activity and antioxidant activity tests were performed to determine the effectiveness of bioconversion according to the dosage and fermentation time.

The study was conducted experimentally with a wholly randomized nesting design, where factor A, namely, the inoculum dose of PaRmYCl (D1, 5%; D2, 10%; and D3, 20% v/w); and factor B, namely fermentation time (W1, 2; W2, 4; and W3, 8 days). Factor B is nested in factor A, and each treatment was repeated 3 times. Fermented products are analyzed for their nutritional content (analysis of proximates, amino acids, fatty acids, enzyme activity, and antioxidant activity). The Duncan Double-Distance Test was performed to determine the difference in effect between treatments.

### Biological test stage of FPW

Biological tests were carried out on selected fermented products from the first stage of the study, using the parameters of digestibility and NR. The digestibility of FPW determined by a consortium of three PaRmYl microbes determined the quality and biological benefits of broiler chickens.

### Research procedure

The number of chickens used was 90 broiler chickens aged 35 days, 45 males and 45 females, with an average weight of 2.17 kg/ek, 30 chickens per treatment, and a coefficient of variation of 2.45%. Chickens were randomly placed in 90 metabolic cages. Feeding was performed *ad libitum* for 1–2 weeks to adjust to fermented foods. Feed treatment was as follows: (1) Non-fermentation with animal testing, rooster; (2) FPW, roosters; and (3) FPW, hens. After acclimatizing to the environment (7 days) and feeding (7 days), at the end of the experiment, feeding was carried out using a single feed in the form of semi-pasta, with up to 120 g of each chicken. After 14 h of feeding, all chickens were slaughtered, and their colons were removed to collect fecal samples.

Chemical analysis was performed in the laboratory by measuring DM, organic matter (OM), nutrient content (CP), and food markers; DM, OM, nutrient content (CP), and dirt markers. The data were used to calculate the indigestibility factor (IF), endogenous loss, and standard ileal digestibility of DM, OM, CP, and NR in broiler chickens using the following equation [28]:


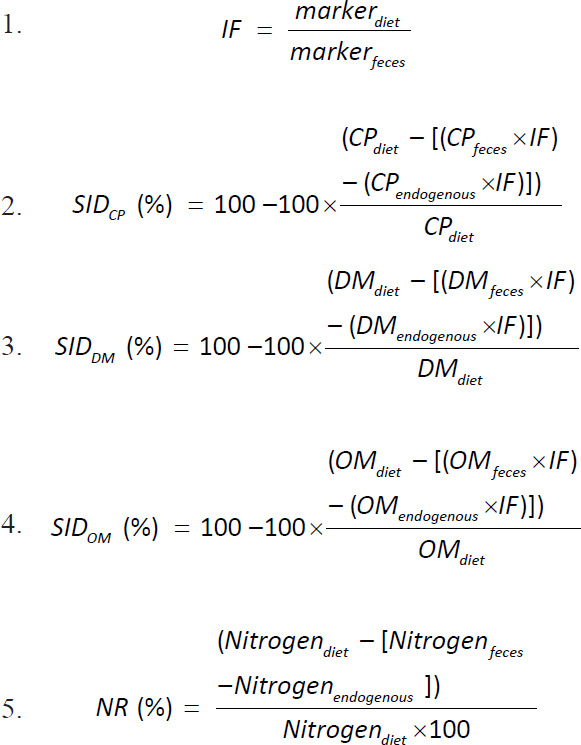


### Statistical analysis

All data were analyzed using a completely randomized design. For the first stage, a nested factorial design was employed, with inoculum dose (5%, 10%, and 20% v/w) as the main factor and fermentation duration (2,4, and 8 days) nested within the dose. Each treatment was replicated 3 times. For the second stage, the biological testing employed a one-way analysis of variance (ANOVA) to evaluate differences among three treatments: non-fermented feed (control), FPW-fed roosters, and FPW-fed hens. Each treatment included 30 replicates. Data normality and homogeneity of variance were assessed using the Shapiro-Wilk test and Levene’s test, respectively. When significant differences were identified (p < 0.05), mean comparisons were conducted using Duncan’s Multiple Range Test to determine pairwise differences. Results are expressed as mean ± standard error of the mean (SEM). All analyses were performed using SAS software (version 9.0, SAS Institute Inc., Cary, NC, USA).

## RESULTS

### Proximate analysis of FPW products by PaRmYl tri-microbial

Nutritional composition of the proximate amounts of unfermented FPW products and fermentation by a consortium of three PaRmYl microbes (*P. aeruginosa, R. microsporus*, and *Y. lipolytica*). Fermentation with 10% inoculum for 4 days significantly increased CP (37.27%) and reduced crude fat content, thereby improving feed quality (Table 1).

**Table 1:** Nutrient composition of FPW via a consortium fermentation of three microbes PaRmYl.

Treatment	CP*	EE*	CF*	Ca	p

	(%)	(%)	(%)	(%)	(%)
NF*	26.05	20.94	2.21	1.50	7.20
D1W1	28.33^b^	18.81^a^	1.86^a^	3.30^c^	7.75^c^
D1W2	31.34^b^	16.47^a^	1.66^a^	5.06^b^	10.81^b^
D1W3	30.75^b^	15.94^a^	1.61^a^	4.88^b^	10.47^b^
D2W1	34.43^b^	15.18^a^	1.36^a^	3.51^c^	7.98^c^
D2W2	37.27^a^	10.51^b^	1.15^b^	5.56^a^	11.46^a^
D2W3	37.13^a^	10.28^b^	1.17^b^	4.85^b^	11.11^b^
D3W1	35.71^a^	13.30^a^	1.27^b^	3.42^c^	7.74^c^
D3W2	37.19^a^	10.37^b^	1.15^b^	5.35^a^	11.36^a^
D3W3	37.15^a^	10.20^b^	1.17^b^	4.82^b^	11.25^b^
SEM**	1.11	1.05	0.09	0.28	0.53

D1=PaRmYl 5% (v/b); D2=PaRmYl 10% (v/w); D3=PaRmYl 20% (v/w); W1=2 days; W2=4 days; W3=8 days. ^abc^Means in the same column/row with different superscript letters differ significantly (p<0.05). *NF=Non-fermentation, CP=Crude protein, EE=Extract ether, CF=Crude fiber, **SEM=Standard error of the mean, FPW=Pangasius fish waste, PaRmYl=*Pseudomonas aeruginosa, Rhizopus microsporus,* and *Yarrowia lipolytica*

Table 1 illustrates that FPW bioconversion results in a variety of nutrient compositions. CP, phosphorus, and crude fiber (CF) content did not differ substantially at 10% and 20% inoculum doses. Nevertheless, the results differed (p < 0.05) compared with 5% inoculum doses for the nutritional content of the product. For 10% inoculum (D2) doses, the 4- and 8-day fermentation times showed significant increases in CP, ether extract (EE), and CF content (p < 0.05) compared with the 2-day fermentation time. FPW had a much higher Ca and P content after 4 days of fermentation (p < 0.05) than after 2 or 8 days. Table 1 shows that EE and CF FPW were lower than NF, indicating an improvement in the quality of fermented fish waste. The increase in the nutritional composition of FPW was also demonstrated by a decrease in crude fat content to 10.28% and crude fiber to 1.15% at a 10% inoculum dose.

### FPW amino acid content of three PaRmYl microbes

Lysine content showed a significant difference (p < 0.05) between inoculum and oral dose treatments, whereas methionine content did not differ significantly. The highest lysine content was observed at a 20% inoculum dose for 8 days but was not significant at a 10% inoculum dose for 4 days. At the same time, the highest cystine content was at a 20% inoculum dose for 8 days presentation of the amino acids lysine and methionine presented as limited amino acids in poultry feed. The sum of essential amino acid (EAA), total amino acids (TAA), and EAA/nonessential amino acid (NEAA) ratios are presented in Table 2.

**Table 2:** Amino acids in Pangasius fish waste before and after bioconversion.

No.	Nutrient essential amino acids (g/100g)	Non-fermented	PaRmYl, D1 (5%, v/w)	PaRmYl, D2 (10%, v/w)	PaRmYl, D3 (20%, v/w)
1	L-Lysine*	1.29	1.55	1.82	1.84
2	L-Methionine*	0.40	0.49	0.48	0.52
3	L-Phenilalanine*	0.95	1.60	2.04	1.77
4	L-Isoleucine*	0.72	1.27	1.61	1.05
5	L-Valine*	0.93	1.52	1.88	1.31
6	L-Arginine	1.78	1.88	2.20	2.59
7	L-Leucine*	1.51	2.19	2.36	2.08
8	L-Threonine*	1.20	2.50	1.98	2.51
9	L-Histidine*	0.40	0.49	0.81	1.53
10	L-Systine*	0.26	0.41	0.55	0.56
11	L-Serine	1.20	1.75	2.12	1.94
12	L- Glutamic acid	2.85	3.73	3.97	3.43
13	L-Alanine	1.91	2.01	2.41	2.31
14	Glicine	3.84	2.88	2.82	4.29
15	L-Asam Aspartat	1.65	2.36	2.57	2.08
16	L-Tirosine	0.55	2.03	2.14	1.14
17	L-Proline	2.23	1.79	1.78	2.74
Total amino acid		23.66	30.46	33.54	33.69
EAA		9.43	13.91	15.73	15.76
NEAA		14.22	16.55	17.82	17.93
EAA/NEAA ratio		0.66	0.84	0.88	0.88

PaRmYl=*Pseudomonas aeruginosa, Rhizopus microsporus,* and *Yarrowia lipolytica,*

* EAA=Essential amino acids, NEAA=Non-essential amino acid

Table 2 shows that fermentation times of 4 and 8 days increase the amino acid content of lysine, cystine, EAA, TAA, and EAA/NEAA ratio compared with the duration of the 2-day fermentation. NEAA-fermented products are highest after 8 days of fermentation compared with 2 and 4 days. PaRmYl, which comes from a consortium of three microorganisms, has an effective inoculum dose of 10% and a fermentation time of 4 days to increase the required amino acid content (EAA) of fermented pangasius waste products. The EAA/NEAA ratio of 0.88 (88%) indicates that the fermented product produces balanced EAAs for poultry feed.

### FPW fatty acid content of three PaRmYl microbes

The FPW fatty acid content analysis results of the three PaRmYl microbes are shown in Table 3.

**Table 3:** Fatty acid content FPW with bioconversion dose and time.

Treatment	ω-3 **	ω-6	ω-9	UFA	SFA	TFA	DHA	EPA	EFA/NEFA (%)

	(%)	(%)	(%)	(%)	(%)	(%)	(%)	(%)	
NF*	0.22	0.72	0.30	5.87	8.25	14.12	0.70	0.07	9
D1W1	0.37^c^	1.38^c^	0.49^b^	6.46^c^	7.66^a^	14.12^c^	1.32^c^	0.08^b^	16
D1W2	0.43^b^	1.46^c^	0.63 ^b^	8.74^c^	6.50 ^a^	15.24^c^	1.54^c^	0.10^b^	17
D1W3	0.45 ^b^	1.44^c^	0.65 ^b^	9.34^c^	5.89 ^a^	15.22^c^	1.55^c^	0.12^b^	17
D2W1	0.42^b^	1.44^c^	0.63^b^	8.73^c^	6.17^a^	14.90^c^	1.82^b^	0.11^b^	17
D2W2	0.54^a^	1.74^b^	0.88^a^	14.59^a^	5.28^b^	19.87^a^	2.16^a^	0.15^a^	16
D2W3	0.53^a^	1.65^b^	0.82^a^	12.65^b^	5.27^b^	17.93^b^	2.22^a^	0.14^a^	17
D3W1	0.29^c^	1.85^b^	0.53^b^	9.85^c^	6.67^a^	16.52^c^	1.59^b^	0.12^b^	16^b^
D3W2	0.52^a^	2.25^a^	0.78^a^	14.72^a^	5.47^b^	20.19^a^	1.61^b^	0.13^b^	18^a^
D3W3	0.53^a^	2.30^a^	0.78^a^	13.83^b^	5.38^b^	19.21^b^	1.86^b^	0.15^a^	18^a^
SEM***	0.03	0.10	0.04	0.96	0.26	0.75	0.10	0.01	0.25

*NF=Non-fermentation, D1=PaRmYl 5% (v/b), D2=PaRmYl 10% (v/w), D3=PaRmYl 20% (v/w), W1=2 days, W2=4 days, W3=8 days, (PaRmCl=*P. aeruginosa, R. microsporus* and *Y. lipolytica*). **ω-3=Omega 3, ω-6=Omega 6, ω-9=Omega 9, UFA=Unsaturated fatty acid, SFA=Saturated fatty acid, TFA=Total fatty acid, EPA=Eicosa pentanoic acid, DHA=decosa-pentanoic acid, EFA/NEFA=Essential fatty acid/nonessential fatty acid. ***SEM=Standard error of the mean. ^abc^Means in the same column/row with different superscript letters differ significantly (p<0.05)

Table 3 shows the production of different fatty acid profiles. In 4 and 8 days of fermentation, DHA at 10% inoculum doses showed significant differences (p < 0.05) compared with 5% and 20% inoculum doses. The fatty acid content of ω-3, ω-9, saturated fatty acids (SFA), and EPA at 10% and 20% inoculum doses showed no significant but significant results (p < 0.05) compared with 5% doses. The content of ω-6 fatty acids, unsaturated fatty acids (UFA), and total fatty acids (TFA) at the 20% inoculum dose was significant (p < 0.05) compared to the 5% and 10% doses.

The 4- and 8-day fermentation times were significant (p < 0.05) compared with the 2-day fermentation time on the fatty acid content of ω-3, ω-6, and ω-9, SFA, DHA, and EPA of the fermented products. The UFA and TFA content of the fermented products after 4 days of fermentation was significantly higher (p < 0.05) compared with the 2-day and 8-day fermentation. This study revealed an increase in the fatty acid profiles of UFAs, TFAs, EPA, and DHA (Table 3) and a slight decrease in SFA content (Table 3). A consortium of three PaRmYl microbes at a dose of 10%–20% with a fermentation time of 4 days is an effective dose and time to increase the content of essential fatty acids in FPW and produce the highest ratio of essential/non-essential fatty acids. The rise in ω-3 and ω-6 content enhances the nutritional value of poultry meat, which benefits both poultry health and consumer nutrition.

### Enzyme and antioxidant activities during FPW bioconversion by three PaRmYl microbes

Enzymatic and antioxidant activities in the bioconversion of three microbes of FPW PaRmYl are shown in Table 4.

**Table 4:** Enzymatic and antioxidant activities of FPW by three microbes (PaRmYl).

Treatment	Protease (U/mL)	Lipase (U/mL)	Antioxidant activity (IC_50_ ppm)
NF*	1.29	0.58	60.84
D1W1	1.36^b^	0.72^b^	50.48^a^
D1W2	1.39^b^	0.84^b^	46.62^a^
D1W3	1.40^b^	0.91^b^	44.31^a^
D2W1	1.42^b^	0.82^b^	45.29^a^
D2W2	1.49^a^	1.21^a^	39.84^b^
D2W3	1.49^a^	1.22^a^	38.03^b^
D3W1	1.45^b^	0.79^b^	43.79^b^
D3W2	1.49^a^	1.14^a^	41.87^b^
D3W3	1.47^a^	1.12^a^	39.23^b^
SEM**	0.02	0.06	1.28

*NF=Non-fermentation, D1=PaRmYl 5% (v/b), D2=PaRmYl 10% (v/w), D3=PaRmYl 20% (v/w), W1=2 days, W2=4 days, W3=8 days, PaRmYl=*Pseudomonas aeruginosa, Rhizopus microsporus,* and *Yarrowia lipolytica*, FPW=Pangasius fish waste. **SEM=Standard error of the mean. ^abc^ Means in the same column/row with different superscript letters differ significantly (p<0.05).

Table 4 shows that the activities of proteases, lipases, and antioxidants have varying values. Enzyme and antioxidant activities at 10% and 20% inoculum doses were not significantly different (p < 0.05) compared with 5% doses. The activities of protease and lipase enzymes in FPW bioconversion at 4- and 8-day fermentation times were significant (p < 0.05) compared with 2-day fermentation. The antioxidant activity in the 8-day fermentation bioprocess was significant (p < 0.05) compared with the 2-day and 4-day fermentations. Good antioxidant activity indicators are indicated by an IC value of 50, whereas the smallest IC_50_ value indicates the highest antioxidant activity. A consortium of three microbes, PaRmYl, at a dose of 10% with a fermentation time of 4 days, is an effective dose and time to produce enzyme and antioxidant activity in bioconverted FPW. FPW, created by a consortium of three microbes, PaRmYl, provides essential nutrients and can be used as a poultry feed supplement.

### Digestibility of FPW by three PaRmYl microbes in broiler chickens

The biological properties of FPW as a feed supplement for chickens were also measured using digestibility tests and NR values (Table 5). FPW tests are carried out on male and female chickens to provide recommendations to broiler chicken breeding companies (parent stock).

**Table 5:** Digestibility nutrient of pangasius fish waste before and after fermentation by a consortium of three microbes PaRmYl in male and female broiler chickens.

Digestibility (%)	Non-fermentation	FPW as a feed supplement		SEM*

		Male	Female	
Dry matter digestibility	67.12±0.54^c^	75.53±0.58^a^	74.37±0.42^b^	0.70
Protein digestibility	63.44±0.35^c^	71.86±0.49^a^	67.61±0.59^b^	0.64
Organic matter digestibility	62.43±0.38^c^	74.09±0.61^a^	72.11±0.36^b^	0.95
Nitrogen retention	67.12±0.54^c^	75.53±0.58^a^	74.37±0.42^b^	0.70

* SEM=Standard error of the mean. ^abc^Means in the same column/row with different superscript letters differ significantly (p<0.05).

Table 5 shows that the digestibility and NR values of fermented products (p < 0.05) were significantly higher than those of non-fermented products and that the digestibility and NR values of roosters (p < 0.05) were considerably higher than those of hens.

## DISCUSSION

### Nutritional composition of FPW as a bioconversion product

The protein content of FPW (28.33%–37.27%) was significantly higher (p < 0.05) compared to the original material (26.05%). The most significant change was 43% of the initial protein content following D2W2 treatment (10% dose, over 4 days). In contrast to fermented products, it is reported that the chemical degradation of tilapia waste in silage products, the degradation of proteins that occur, can reduce the protein content and TAA, as well as in silage fermented by *L. plantarum* with the addition of molasses during the processing time of 9 days [29]. In this study, an increase in the nutritional composition was caused by three types of microbes used in bioconversion, which could improve the enzymatic ability of primary metabolites. This can be explained by the fact that proteases are specific enzymes; thus, using a variety of microbes in the fermentation process can help hydrolyze different protein molecules [24]. The fermentation process of the three types of microbes enhances CPs by the action of decarboxylation, transaminases, and amino peptidases as regulatory enzymes that work simultaneously [15]. *P*. *aeruginosa* is a bacterium with high proteolytic activity that can produce extracellular peptidase enzymes in fermented sausages to hydrolyze sarcoplasmic proteins [30, 31]. In addition, the volatile acids and lactic acids produced by *P. aeruginosa* increase protein degradation activity and have higher activity than *L. plantarum* 8014 and *B. subtilis* [17, 32].

Table 1 shows nutrient composition of FPW through a consortium fermentation of three microbes PaRmYl. The increase in the CP, calcium, and phosphorus content of FPW by three microbes PaRmYl, at a dose of 10% (v/w) over 4 days (43%, 271%, and 59%, respectively. In contrast to the results of a study on chemical fish silage [33], the total protein content of processed products was generally lower, and the results of fermentation using one type of microbe in tilapia waste showed a decrease in total protein from 37.67% to 35.84% [30]. Similarly, the fermentation of fish waste by *L. plantarum* ATCC 8014, *P. aeruginosa*, and *R. microsporus* shows the protein hydrolysis process that occurs during fermentation, so there is a decrease in the CP content of fish waste with a longer fermentation process [34].

The increase in protein products in this study can be explained by the statement [35] that biosynthetic and catabolic processes work in tandem with the dosage of probiotic products in fermentation, which are used as amino acid precursors to produce essential nutrients (EAAs). Catalic precursors can also function as biosynthetic precursors [36]. Decarboxylation of the protein skeleton, which is intended as a precursor to amino acids for biosynthesis, increases the number of amino acids and N compounds, thereby increasing protein content [37].

The growth of microbial cells forms a single protein cell. Proteases of the three types of microbes allow for a complementary mixture of various specific enzymes. Among the complementary effects are the actions of transaminase enzymes on microbes and amino peptidases, increasing their total protein levels. In addition, according to Sankian *et al*. [21] and Vidotti *et al*. [29], regulatory enzymes also work during fermentation in biological processes. Allosteric enzymes can detect and respond to the concentration of substances in species as (regulators), including the OM of pangasius waste.

Table 1 also shows a decrease in crude fat and crude fiber content (inoculum *Y. lipolytica* at a dose of 10% over 4 days). Microbes use the fat in Pangasius waste to multiply during the exponential phase. Lepase, under certain conditions, can catalyze the synthesis of reactions, such as esterification, transesterification (interesterification, acidosis, and alcohol lysis), amino lysis (amide synthesis), and lactonization (esterification of esterified intermolecular esterification) [38].

The decrease in crude fiber content during the fermentation process is due to microbial activity, which breaks down bonds in fish waste materials. According to the Wu *et al*. [39], the microbiota converts indigestible fiber into short-chain fatty acids (SCFAs; acetate, propionate, and butyrate), which have anti-inflammatory properties in chicken immune cells both *in vitro* and *in vivo*, thereby beneficial for poultry farming. The percentage of crude fiber is vital in animal feed because it affects the rate of feed-crossing mucosal function and acts as a substrate for intestinal bacteria, thereby affecting digestive performance and health. Reducing crude fat is more beneficial for storing the product when fermentation plays a preservation function.

### Amino acid composition and EAA ratio in FPW

Table 2 shows the results of amino acid analysis of FPW at various doses, which shows an improvement compared with no NF fermentation. The PaRmYCl microbial consortium increased the content of EAAs at 10% and 20% inoculum doses. The increase in amino acid content is caused by the activity of proteases during the catabolization of fish waste. Extracellular proteases are essential in the hydrolysis of protein substrates into amino acids [7]. *P. aeruginosa* can increase the levels of asparagine, proline, lysine, valine, and threonin [11, 40]. *R. microsporus* is reported to be able to hydrolyze sarcoplasmic proteins and increase free amino acids such as glutamine and alanine [10]. In this study, the content of several types of EAA in fermented products increased, such as phenylalanine (2.04%), isoleucine (1.61%), valine (1.88%), leucine (2.36%), threonine (1.98%), and histidine (0.81%).

The ratio of essential and NEAA was higher before fermentation. This suggests that fermentation using a consortium of *P. aeruginosa, R. microsporus*, and *Y. lipolytica* microbes can improve the content of EAAs by changing the EAA/NEAA ratio from 0.66 to 0.88 at 10% and 20% inoculum doses. The three-microbial inoculum of PaRmYl at a dose of 20% for 8 days increased the content of lysine (57%), cystine (143%), NEAA (36%), and the highest TAA (52%) (Table 2). The increase in EAA (79%) and EAA/NEAA ratio (44%) was highest at a dose of 10% over 4 days.

In the results of this study, the overall content of EAAs showed an increasing trend. In contrast to a previous study by Vidotti *et al*. [29], the amino acid composition of fish silage through the fermentation of tilapia waste using *R. microsporus* showed an increase in NEAA (histidine, threonine, and serine) levels. At the same time, EAA (valine, isoleucine, and leucine) decreased. During fermentation, a chemical reaction occurs between the amino group and aldehydes in the amino acid [40], and hydrolysis by protease produces various amino acids and peptides. With the rise and fall of amino acid levels, there is a balance of amino acids in FPW products, indicating that the performance of the consortium of three microbes, PaRmYl, is very synergistic in producing proteases. Regulatory enzymes during fermentation, resulting in a decrease in NEAA and an increase in EAA. This result is due to gluconeogenesis from glucogenic amino acids [41].

During fermentation, decarboxylation, transaminase, and amino peptidase reactions occur [7]. These improvements make FPW products the most widely available source of amino acids for protein synthesis. According to Venegas-Ortega *et al*. [19], peptide synthesis requires a medium that promotes bacterial growth. Nonetheless, the protein content is adequate for peptide synthesis through enzymatic activity. FPW is a protein matrix that can replace microbial culture media at a lower cost. It has been shown that NEAA is used to feed microbes and is then converted to EAA.

### Fatty acid profile

In general, the fatty acid content of FPW was increased (Table 3), indicating the activity of the lipase enzyme PaRmYl produced by the three microbes. The improvement of the fatty acid profile (Table 4) showed that the fatty acids fermented by the three PaRmYCl microbes, at a dose of 10% inoculum during 4 days of fermentation, increased the content of ω-3, ω-9, UFA, total fatty acids (TFA), and EPA by 140%, 193%, 149%, 41%, and 114%. The highest content of ω-6 fatty acids, DHA, and the UFA/SFA ratio was produced at a 20% inoculum dose for 4 days, with percentage increases of 214%, 129%, and 100%, respectively. Supplementation of *Y. lipolytica* as a probiotic can modulate lipid accumulation in high-fat feeds to produce essential fatty acids oleic acid 4.65 mg linoleic acid 18.59 mg with a total increase in fatty acids 13.74 mg [42].

Lipase catalyzes the total or partial hydrolysis of fats and oils. According to Ghaly *et al*. [7], the activity of lipase during fermentation can reduce the fat content of FPW. This is supported by the fact that the waste material of the pangasius hypothalamus still contains UFAs, and the fatty acid profiles of different body parts did not show significantly different results [23]. Decreased levels of linolenic acid can increase the levels of the fatty acid derivative ω-3, usually synthesized from linolenic acid. UFA can also be obtained from plant food sources such as EPA and DHA [43].

Table 3 presents the decreased SFA content in FPW. Using a three-microbial inoculum dose of PaRmYl by 10% during the 4-day fermentation time reduced SFA, with the highest percentage of 36%. The lipolytic activity of microbes can release free fatty acids, diacylglycerol, monoacylglycerol, and glycerol [44]. Palmitic and stearic acids (SFA) can be denatured as oleic and linoleic acids through lipid hydrolysis by lipase. Thus, oleic and linoleic acids in FPW are increased more than other fatty acids. *Y. lipolytica* with *R. microsporus* and *P. aeruginosa* in this study, as a consortium of microbes, in addition to increasing the protein and amino acid content, also succeeded in increasing the fatty acids ω-3 and ω-6 as a necessary nutritional supplement for poultry.

The proteolytic and biopolitical activity of PaRmYl microbes contributes to increased protein hydrolysis and lipid conversion, thereby improving the profiles of amino acids and fatty acids. The results of this study indicate that fermentation with consortium microbes is more effective than that with single microbes. The advantages of fermentation with a consortium of microbes, one of which is converting SFA into unsaturated ones, for the efficiency of poultry feed and meat quality.

### Enzymatic and antioxidant activities

The protease, lipase, and antioxidant activities at 10% and 20% inoculum doses were not significantly different from those at 5% inoculum doses. Table 5 presents the increase in FPW enzyme activity. The fermentation times of 4 and 8 days were significantly compared with the 2 days of fermentation for the activity of protease and lipase enzymes. The three-microbial inoculum PaRmYl at a 10% inoculum dose for 8 days increased the activity of protease enzymes, lipases, and antioxidants by 16% and 37%. According to Lindgren and Refai [33], the enzyme activity in feed added with a pure exogenous MOS inoculum has an optimal limit of 0.8%.

In contrast, the activity of lipase enzymes in fish waste increased from 0.58% to 1.21%. Although the increase was relatively high, lipase activity in FPW treatment was lower than protease activity (Table 4). This causes the fatty acids produced from FPW by the three probiotic microbes, PaRmYl, to become smaller than the amino acids synthesized. Protein, fat, SFA, and UFA nutrients in FPW are potential substrates for producing amino acids and essential fatty acids through fermentation. Bacteria with proteolytic and biopolitical properties can produce proteases, peptidases, and lipases. These enzymes can degrade protein and fat components into amino acids, peptides, and fatty acids [36].

Optimal microbial growth occurs in a nutrient-rich environment. The substrate for microorganisms functions enzymatically to produce secondary metabolites, such as enzymes [45]. The results showed the highest protease enzyme activity at a 10% inoculum dose during 4 days of fermentation. This indicates a low concentration of secondary metabolite products that inhibit microbial growth. According to Youran *et al*. [27], the protease activity of *P. aeruginosa* increases rapidly with fermentation time (5494.1 U/g at 48 h) in chickpea medium (365 organic, Heyday Canning Co., and Cento, United States). The fermentation process in expanding the substrate causes the nutrients in the material to decrease as the fermentation period increases. The mechanism by which enzymes work is that they function precisely on the substrate, which means that if there is no substrate available for the enzyme, there will be no activity. This parameter indicates that the substrate and enzyme molecules will mix at the active sites until all active sites have been used. In this situation, the enzyme is at its peak activity.

Table 4 also shows the anti-oxidant activity of all treatments. The antioxidant activity in the 8-day fermentation bioprocess was significantly higher (p < 0.05) compared to 2-day and 4-day fermentations. The smallest IC_50_ value indicates the highest antioxidant activity. Antioxidant molecules play an essential role in maintaining the balance of the gastrointestinal microbiome by modulating oxidative stress [38, 46]. The mechanism of antioxidant activity is that pangasius meat protein hydrolysate contains hydrophobic amino acids formed during fermentation, has considerable free radical activity, and can protect against oxidation by providing protons to free radicals [22, 39].

A lower value of antioxidant activity (IC_50_ ppm) indicates excellent anti-oxidant activity (practical). Evaluating the antioxidant properties of isolated bacterial strains based on bacterial suspension capability in DPPH and free radical ABTS tests, the *Y. lipolytica* strain significantly decreased lipid peroxidation with increased enzyme activity [32]. *P. aeruginosa* showed the lowest DPPH radical activity (49.34%) [27].

### FPW digestibility in broiler chickens

Table 5 shows the digestibility and NR values of FPW by three PaRmYl microbes in broiler chickens. Biological tests on broiler chickens showed increased digestibility of dry, protein, and OM (Table 5). In the results of this study, FPW significantly (p < 0.05) increased digestibility compared with the absence of fermentation. Roosters digested FPW more efficiently than hens, with DM, protein, and OM digestibility rates of 75.53%, 71.86%, and 74.09%, respectively. Fermented feed is used in poultry production because it improves the microbial ecology related to intestinal function and antioxidant status.

Microorganisms in the fermentation process produce beneficial nutritional products using microbial enzymes and affect poultry by being involved in physiological processes such as digestion, nutrient absorption, energy balance, and modification of many possible bioactive metabolites. According to Shabani *et al*. [47], fish silage made with a single microbe, *L. plantarum*, can increase digestive enzyme activity due to the concentration of organic acids, especially lactic acid, and in broiler chicken feed lowers the pH level in the digestive tract while increasing the content of SCFAs. In addition, Wang *et al*. [48] found that enzyme supplementation improved feed digestibility in broiler chickens by decreasing jejunal viscosity.

Mixed microbial cultures provide interesting and unexplored advantages over standard monoculture-based techniques. FPW improves the digestibility of DM, OM, and protein digestibility in roosters and females by up to 70% compared with the original material. The NR value of FPW products produced by the three microorganisms PaRmYl was higher (75.53%) than that of FPW without fermentation (67.12%). This suggests that nitrogen intake from food is superior for use as a protein biosynthesis substrate. FPW products differ from their original ingredients (non-fermented NF) in terms of nutrient composition, protein and amino acid content, and digestibility. Bioconversion is an effective strategy to improve the nutritional characteristics of feed.

FPW, as shown in Table 1–3, produces value-added products in the form of essential nutrients. FPW can produce fish protein concentrate, fish oil, and enzymes as feed additives for protein supplements and other added value [34, 43, 44]. Amino acids, increased digestive enzyme activity, and fatty acids all contribute to the balance of the gut ecosystem and have been shown to improve the developmental performance of chickens fed fermented pangasius fish feed. This is corroborated by the findings of peptide assessment from fermented fish, which showed antioxidant, anti-inflammatory, anti-pathogen, and anti-nutrient effects while improving digestibility, taste, nutrient content, texture, aroma quality, and other characteristics [12].

Using the PaRmYl consortium because of this study will increase synergy and the expected nutritional results. Mixed strain cultures (*L. plantarum* 120, *L. plantarum* 145, and *P. pentosaceus* 220) reduced fermentation time and improved the quality of all fish-based products [12]. Like fermentation with fungi, fermentation with LAB can suppress the growth of spoilage microbes and improve organoleptic quality. *R. microsporus* has the potential as an anti-pathogen by suppressing gram-negative bacteria such as *E. coli*. Modulating the microbiome of food components and additives can affect poultry by participating in digestion, nutrient absorption, mucosal immune response formation, energy homeostasis, and the synthesis or modulation of several potential bioactive metabolites [39].

From a poultry nutrition perspective, lysine, cystine, and methionine are EAAs for monogastric animals, including chickens [10]. The FPW nutrients in Table 2 represent potential sources of EAAs that meet Food and Agriculture Organization standards [29]. An aspect considered in poultry feed formulas is the ratio of amino acids from animals to plants, with a ratio of 1/3 of the TAAs in the feed. FPW by three microbes, PaRmYl, can balance the nutrients that poultry require. Therefore, this product can be used as a feed supplement for poultry. It is recommended that the potential of improving the fermentation process for industrial use or testing FPW in other livestock be explored.

## CONCLUSION

The study introduces an innovative and cost-effective method for utilizing fish waste as a sustainable poultry feed supplement. The use of a microbial consortium enhances the synergistic effects on nutrient profiles, digestibility, and antioxidant properties, offering a scalable solution for feed production while addressing environmental sustainability challenges. However, the study primarily focuses on broiler chickens, limiting its generalizability to other poultry species or livestock. Long-term effects of FPW on poultry health, productivity, and meat quality were not evaluated, and industrial scalability, along with cost-benefit analysis of large-scale FPW production requires further exploration.

Future research should investigate the impact of FPW on other poultry species, livestock, and aquaculture systems to validate its broader applicability. Longitudinal studies are needed to assess the long-term effects of FPW on poultry health, growth, and productivity. Optimization of fermentation processes for industrial-scale production and economic and environmental feasibility studies will further support the commercialization of FPW. In addition, its potential as a dietary supplement to enhance the nutritional quality of poultry meat for human consumption should be explored.

FPW bioconversion using PaRmYl offers a sustainable and nutritionally effective solution for poultry feed. Further research can extend its applicability and contribute to the development of sustainable animal husbandry practices.

## DATA AVAILABILITY

All the data generated or analyzed during the study are included in the article.

## AUTHORS’ CONTRIBUTIONS

AA: Designed and conducted the study, including fermentation collection and investigation and feed testing, statistical analysis, and drafted and edited the manuscript. TW and DR: Chemical analysis of feed, investigated enzyme activity and antioxidant activity, assessed digestibility, and performed statistical analysis. KH: Conducted research, including bioconversion of FPW and proximate analysis of FPW products by PaRmYl tri microbial. TW: Co-ordinated and provided guidance on the study and reviewed the manuscript. All authors have read and approved the final manuscript.
